# Prevalence of HIV and syphilis co-infection and associated factors among non-commercial men who have sex with men attending a sexually transmitted disease clinic in Shenzhen, China

**DOI:** 10.1186/s12879-017-2187-1

**Published:** 2017-01-18

**Authors:** Wenjie Dai, Zhenzhou Luo, Ruiwei Xu, Guanglu Zhao, Dan Tu, Lin Yang, Feng Wang, Yumao Cai, Lina Lan, Fuchang Hong, Tubao Yang, Tiejian Feng

**Affiliations:** 1 Department of Epidemiology and Health Statistics, Xiangya School of Public Health, Central South University, Changsha, Hunan China; 2 Shenzhen Center for Chronic Disease Control, Shenzhen, Guangdong China; 3 Shenzhen Nanshan Center for Chronic Disease Control, Shenzhen, Guangdong China; 4 Graduate School, Shenzhen University, Shenzhen, Guangdong China

**Keywords:** HIV, Syphilis, Co-infection, Non-commercial men who have sex with men, Sexually transmitted disease clinic

## Abstract

**Background:**

Although HIV and syphilis co-infection has been frequently observed in men who have sex with men (MSM), only few studies have focused on it. Different subgroups of MSM might exhibit heterogeneous HIV and syphilis risk profiles, indicating that interventions for HIV and HIV-related co-infections may vary with different subgroups of MSM. However, no previous study has investigated HIV and syphilis co-infection among non-commercial MSM (ncMSM) attending a sexually transmitted disease (STD) clinic. Therefore, this study aimed to explore the prevalence of HIV and syphilis co-infection and associated factors among ncMSM attending an STD clinic in Shenzhen, China.

**Methods:**

NcMSM attending the STD clinic of Shenzhen Center for Chronic Disease Control were recruited in this cross-sectional study every Monday between March 2013 and August 2015 using a site based convenience sampling method. An anonymous questionnaire was used to collect data regarding socio-demographic characteristics, risky sexual behaviors and HIV-related knowledge. Blood samples were collected to perform HIV and syphilis tests.

**Results:**

Totally 533 participants were enrolled in this study and the prevalence of HIV and syphilis co-infection among them was 13.13%. Multivariable analyses indicated that having lived in Shenzhen for less than one year (aOR = 2.80, 95% CI = 1.30–6.05), having first anal sexual intercourse before the age of 18 (aOR = 2.78, 95% CI = 1.29–5.89), having 3 to 5 anal sexual partners in the past six months (aOR = 2.54, 95% CI = 1.19–5.40), playing exclusively receptive (aOR = 6.87, 95% CI = 3.02–15.61) or both insertive and receptive (aOR = 3.65, 95% CI = 1.64–8.09) roles in anal sexual intercourse and not always using condom in anal sexual intercourse (aOR = 2.13, 95% CI = 1.08–4.19) were associated risk factors for HIV and syphilis co-infection, relative to the non-infected ncMSM. Compared with the mono-infected ncMSM, associated risk factors for the co-infection were being unmarried (aOR = 2.47, 95% CI = 1.03–5.89) and playing exclusively receptive role (aOR = 2.44, 95% CI = 1.04–5.73) in anal sexual intercourse.

**Conclusions:**

HIV and syphilis co-infection is quite prevalent among the study participants in Shenzhen. Integrated and intensified intervention strategies, specifically targeting at the non-infected and mono-infected ncMSM attending the STD clinic, are needed to reduce HIV and syphilis co-infection. Most importantly, non-infected and mono-infected ncMSM attending the STD clinic with the aforementioned associated risk factors should be given special concern.

## Background

Over the past few decades, the resurgence of syphilis has been reported worldwide, especially among men who have sex with men (MSM) [1–3]. For example, the prevalence of syphilis was 2.1 cases per 100,000 persons in 2000 in the United States, which increased to 3.0 cases per 100,000 persons in 2005 and, correspondingly, the cases of syphilis increased from 5976 to 8724, of which MSM accounted for 86% [4, 5]. In the United Kingdom, a substantial increase in the cases of syphilis between 1998 and 2003 was observed, with a 25-fold increase seen in MSM (from 43 to 1028 cases) [6]. Similarly, in China, cases of syphilis have been increasing. Syphilis was almost eradicated in China in the 1960s [7] but now it has re-emerged as one of the most common sexually transmitted diseases (STDs) among MSM [8], with the prevalence increasing from 6.8% in 2003–2004 to 13.5% in 2007–2008 [9].

Along with the resurgence of syphilis in China was a rapidly expanding infection of human immunodeficiency virus (HIV) among MSM [9, 10]. According to a meta-analysis involving seventy-one eligible studies, the prevalence of HIV among MSM in China increased from 1.3% in 2003–2004 to 4.7% in 2007–2008 [9], and simultaneously, the prevalence of HIV and syphilis co-infection increased from 1.4% in 2005–2006 to 2.7% in 2007–2008. Notably, HIV and syphilis co-infection has been frequently observed in MSM worldwide [11, 12]. It is well-established that the increase in cases of HIV has played an important role in the resurgence of syphilis, which, in turn, provides a favorable environment for HIV transmission [11, 13]. Previous studies have indicated that syphilis infection can put an individual at three to five-fold higher risk for HIV infection [14] and HIV infection can also greatly increase the risk for primary or secondary syphilis infection [15]. In this regard, integrated HIV and syphilis surveillance and intervention strategies for MSM are needed urgently [16].

However, though evidence has shown that HIV and syphilis co-infection has been frequently observed in MSM [11, 12], studies exploring HIV and syphilis have mainly focused on either of them [17, 18], but rarely on their co-infection [16]. The mechanism of HIV and syphilis co-infection is complex and remains incompletely understood, despite both of them being commonly transmitted via sex and mother to child or fetus [5]. Recently, one of few relevant studies found that, the prevalence of HIV and syphilis co-infection among MSM in the seven cities of China (Nanjing, Jinan, Chongqing, Guangzhou, Harbin, Yangzhou and Suzhou) was 2.6% in 2008 and the co-infection was associated with some socio-demographic characteristics and risky sexual behaviors, such as age, educational level and unprotected anal sexual intercourse [16]. However, the overall MSM population enrolled in that study might be heterogeneous, although relatively big and representing MSM in a wider range of cities in China. It is well understood that different subgroups of MSM might be heterogeneous in their HIV and syphilis risk profiles. For example, the HIV and syphilis risk profiles for money boys (MBs), also known as male sex workers, were quite different from those of non-commercial MSM (ncMSM) [19]. Zhao et al. found that, MBs differed from ncMSM not only in the prevalence of HIV but also in some sexual behaviors [20]. Also, Hong et al. found that socio-demographic characteristics and risky sexual behaviors varied greatly among MSM recruited from different settings (gay bar, gay sauna and STD clinic) [21]. The findings of these studies indicate that interventions for HIV, as well as HIV-related co-infections, may vary with different subgroups of MSM with respect to the targeted risk factors. Currently, the prevalence of HIV and syphilis co-infection and associated factors among ncMSM attending the STD clinic remain unclear.

Shenzhen, located in Guangdong province of southern China, just north of Hong Kong, is the first special economic zone in China [22]. Similar to large cities with well-established populations of MSM like California in USA and Beijing in China, the rapid growth of the MSM population in Shenzhen over the past two decades has drawn attention to Shenzhen as an ideal city to explore HIV-related infections [22]. In this study, ncMSM attending an STD clinic in Shenzhen were recruited to explore the prevalence of HIV and syphilis co-infection and associated factors. Associated factors measured in this study included not only socio-demographic characteristics and risky sexual behaviors, which have been reported to be associated with HIV and syphilis among MSM in many previous studies [18, 23, 24], but also HIV-related knowledge. An investigation of an association of HIV-related knowledge with HIV and syphilis co-infection in this study is very important because HIV-related knowledge has been shown to have a great effect on one’s sexual behaviors, thus playing an important role in HIV infection, as well as in HIV-related co-infections, but only a few studies have measured it among MSM [25–27]. To the best of our knowledge, this might be the first study to explore the prevalence of HIV and syphilis co-infection and associated factors among ncMSM, as well as MSM attending the STD clinic.

## Methods

### Participants

The STD clinic of Shenzhen Center for Chronic Disease Control (CCDC), which has similar characteristics with many other STD clinics in the same city, is one of the largest STD clinics in Shenzhen. MSM attending the STD clinic of Shenzhen CCDC were from many different areas throughout Shenzhen, thus may represent the overall MSM population from all STD clinics in Shenzhen to a large extent. Due to the hard-to-reach nature of the MSM population, participants of this study were selected from those attending the STD clinic of Shenzhen CCDC using a site based convenience sampling method. According to the research programme for exploring the prevalence and associated factors of STDs provided by the Chinese Center for Disease Control and Prevention (CDC), the minimum sample size for a study targeting at a MSM population is 350 (http://www.docin.com/p-658439166.html&dpage=1&key=%E6%80%A7%E7%97%85%E6%80%8E%E4%B9%88%E6%B2%BB). Given the average number of MSM attending the STD clinic of Shenzhen CCDC per day, recruitment of participants in this study was carried out every Monday between March 2013 and August 2015 to make sure that the sample size of this study met the requirement recommended by the Chinese CDC. Supported by the Project of the National Natural Science Foundation of China, free HIV and syphilis tests and consultations were provided for the participants. The inclusion criteria for participants were: (1) biologically male; (2) self reported having had anal sexual intercourse with one or more biologically male partners in the previous year; (3) at least 18 years old; (4) not having paid or having been paid for anal sexual intercourse in the past six months; and (5) willing to participate in the study. MSM with any mental diseases or severe medical illness were excluded. Besides, MSM with a history of drug use were also excluded since they might have been co-infected with HIV and syphilis via injection-drug-use practices and this study focused on HIV and syphilis co-infection via sexual transmission.

### Data collection

Well qualified investigators who had either studied in a medical school or worked for the Shenzhen CCDC were appointed to collect data. Unified training, guided by a written investigation manual, was provided to the investigators prior to data collection. The investigators conducted face-to-face interviews with participants using an anonymous questionnaire to obtain information on socio-demographic characteristics, risky sexual behaviors and HIV-related knowledge.

### Measurements

#### Socio-demographic characteristics

The following socio-demographic variables were measured in this study: age (categorized as “<25 years” and “≥25 years” [28–30]), ethnicity (categorized as “Han” and “other minorities” [26]), marital status (categorized as “married” and “unmarried” [31]), educational level (categorized as “≤junior middle school” and “>junior middle school” [30, 32]), socioeconomic disconnection (neither currently employed nor a student [33]), monthly income (categorized as “≤3000 RMB”, “3001-5000 RMB” and “>5000 RMB” [31, 34]) and length of stay in Shenzhen (categorized as “<1 year”, “1-2 years” and “>2 years” [31, 35]).

#### Risky sexual behaviors

In line with some previous studies [36, 37], risky sexual behaviors measured in this study included having first anal sexual intercourse before the age of 18, not having vaginal sexual intercourse, not having a fixed homosexual partner (defined as not having an anal sexual relationship with the same homosexual partner for at least six months), having multiple (>3) anal sexual partners in the past six months, playing exclusively receptive or both insertive and receptive roles in anal sexual intercourse and not always using condom in anal sexual intercourse in the past six months.

#### HIV-related knowledge

According to the evaluation framework for preventing and controlling HIV in China enacted by the State Council of China (http://www.docin.com/p-854479849.html&key), eight HIV-related questions, which have been widely used in China, were applied to evaluate the participants’ HIV-related knowledge [38, 39]. The questions were structured as follows: could mosquito bites transmit HIV? Do people infected with HIV look healthy? Could blood transfusion or use of blood products transmit HIV? Could sharing a meal with a person infected with HIV transmit HIV? Could delivery transmit HIV to the born child? Could sharing a needle with someone who was infected with HIV transmit HIV? Could having sex with a fixed sexual partner reduce the risk of contracting HIV? And, could correct use of a condom each time you have sex transmit HIV? The score of one point was assigned to each correct answer and zero to each wrong answer so that the number of correct answers was equal to the total score, which ranged from 0 to 8 points. According to the evaluation framework, individuals with at least 6 points were regarded as having a comprehensive knowledge of HIV [40].

#### HIV and syphilis tests

HIV and syphilis tests were performed on blood samples of the participants. The tests were guided by the standardized laboratory procedures provided by the Chinese CDC. The enzyme-linked immunosorbent assay (ELISA) (Wantai Biotech Inc, Beijing, China) was used for screening HIV and Western blot (MP Diagnostics, Singapore) was used to confirm the HIV screening results. Syphilis was screened using ELISA (Lizhu Biotech Inc, Zhuhai, China) and then the syphilis screening results were confirmed by toluidine red unheated serum test (TRUST) (Rongsheng Biotech Inc, Shanghai, China). Participants were diagnosed as HIV positive if both ELISA and Western blot showed positive on their blood samples. Similarly, participants with ELISA positive and TRUST positive on their blood samples were diagnosed as syphilis positive. According to the results of HIV and syphilis tests, participants were categorized as the co-infected group (both HIV and syphilis positive), the mono-infected group (either HIV positive or syphilis positive but not both) and the non-infected group (neither HIV positive nor syphilis positive).

#### Data analyses

Statistical analyses were performed in SPSS 19.0 (IBM Corp, Armonk, NY). All statistical tests were 2-tailed and a *P* value of less than 0.05 was considered as significant. Categorical variables were described by frequency counts and corresponding percentages (%), while continuous variables were described by means and corresponding standard deviations (SD). Univariable logistic regression analyses were performed to identify the bivariate relationships between the co-infection of HIV and syphilis, and socio-demographic characteristics, risky sexual behaviors, and HIV-related knowledge. Multivariable logistic regression analyses were used to determine the independent contributions of each associated variable to HIV and syphilis co-infection by simultaneously entering all the associated variables into the logistic regression model (including socio-demographic characteristics, risky sexual behaviors and HIV-related knowledge). Co-infected individuals were compared with non-infected individuals and then with mono-infected individuals. Crude odds ratio (OR) and adjusted OR (aOR) with corresponding 95% confidence interval (CI) were, respectively, obtained from univariable and multivariable logistic regression analyses for each associated variable in order to show the direction and strength of the association.

## Results

### Characteristics of the participants

A total of 533 eligible participants with mean (SD) age of 32.1 (8.4) years were enrolled in this study. Table 1 displays the characteristics of the study participants. Among the 533 respondents, 499 (93.6%) were Han ethnicity, 129 (24.2%) were married, 160 (30.0%) had attended at most junior middle school and 52 (9.8%) were socio-economically disconnected. Also, 354 (66.4%) reported having had vaginal sexual intercourse, 368 (69.0%) had a fixed homosexual partner and 94 (17.6%) had first anal sexual intercourse before the age of 18. Besides, 214 (42.5%) reported always using condoms in anal sexual intercourse in the past six months and 378 (70.9%) scored six or more regarding HIV-related knowledge.

**Table 1:** Characteristics of the study participants (*n* = 533)

Variable	Category	Number	Percent (%)
Age (year)			
	<25	93	17.4
	≥25	440	82.6
Ethnicity			
	Han	499	93.6
	Other minorities	34	6.4
Marital status			
	Married	129	24.2
	Unmarried	404	75.8
Educational level			
	≤Junior middle school	160	30.0
	>Junior middle school	373	70.0
Socioeconomic Disconnection			
	Yes	52	9.8
	No	481	90.2
Monthly income (RMB)			
	≤3000	211	39.6
	3001-5000	183	34.3
	>5000	139	26.1
Length of stay in Shenzhen (year)			
	>2	183	34.3
	1-2	73	13.7
	<1	277	52.0
Having vaginal sexual intercourse			
	Yes	354	66.4
	No	179	33.6
Age at first anal sexual intercourse (year)			
	≥18	439	82.4
	<18	94	17.6
Having a fixed homosexual partner			
	Yes	368	69.0
	No	165	31.0
Number of anal sexual partners in the past 6 months			
	≤2	217	40.7
	3-5	150	28.1
	>5	166	31.2
Roles in anal sexual intercourse^a^			
	Insertive	212	41.1
	Receptive	123	23.8
	Both	181	35.1
Always using condom in anal sexual intercourse in the past 6 months^a^			
	Yes	214	42.5
	No	289	57.5
HIV-related knowledge			
	<6	378	70.9
	≥6	155	29.1

^a^Data may not add up to 533 because of missing data

### Prevalence of HIV and syphilis co-infection

According to the results of HIV and syphilis tests, there were 70 participants in the co-infected group, 148 in the mono-infected group (59 with HIV only and 89 with syphilis only) and 315 in the non-infected group, indicating that the prevalence of HIV, syphilis and their co-infection among the study participants was 24.2% (129/533), 29.8% (159/533) and 13.13% (70/533), respectively.

### Factors associated with HIV and syphilis co-infection relative to non-infection

Univariable logistic regression analyses indicated that having lived in Shenzhen for less than one year (OR = 2.42, 95% CI = 1.27–4.62), having first anal sexual intercourse before the age of 18 (OR = 2.53, 95% CI = 1.38–4.66), having 3 to 5 anal sexual partners in the past six months (OR = 2.26, 95% CI = 1.21–4.19), playing exclusively receptive (OR = 6.78, 95% CI = 3.27–14.07) or both insertive and receptive (OR = 2.84, 95% CI = 1.39–5.78) roles in anal sexual intercourse and not always using condom in anal sexual intercourse (OR = 2.46, 95% CI = 1.38–4.38) were associated with HIV and syphilis co-infection, relative to the non-infected individuals. Multivariable logistic regression analysis indicated that, compared with the non-infected individuals, having lived in Shenzhen for less than one year (aOR = 2.80, 95% CI = 1.30–6.05), having first anal sexual intercourse before the age of 18 (aOR = 2.78, 95% CI = 1.29–5.89), having 3 to 5 anal sexual partners in the past six months (aOR = 2.54, 95% CI = 1.19–5.40), playing exclusively receptive (aOR = 6.87, 95% CI = 3.02–15.61) or both insertive and receptive (aOR = 3.65, 95% CI = 1.64–8.09) roles in anal sexual intercourse and not always using condom in anal sexual intercourse (aOR = 2.13, 95% CI = 1.08–4.19) were associated risk factors for HIV and syphilis co-infection (Table 2).

**Table 2:** Logistic regression analyses of factors associated with HIV and syphilis co-infection (compared with the non-infected individuals)

Variable	Category	HIV and syphilis infection (%)		Crude OR (95% CI)	Adjusted OR (95% CI)
		Co-infected group	Non-infected group		
Age (year)					
	<25	9 (14.5)	53 (85.5)	1	1
	≥25	61 (18.9)	262 (81.1)	1.37 (0.64–2.93)	1.82 (0.71–4.64)
Ethnicity					
	Han	64 (17.8)	296 (82.2)	1	1
	Other minorities	6 (24.0)	19 (76.0)	1.46 (0.56–3.80)	2.00 (0.65–6.15)
Marital status					
	Married	13 (14.9)	74 (85.1)	1	1
	Unmarried	57 (19.1)	241 (80.9)	1.35 (0.70–2.59)	1.64 (0.69–3.89)
Educational level					
	≤Junior middle school	24 (22.2)	84 (77.8)	1	1
	>Junior middle school	46 (16.6)	231 (83.4)	0.69 (0.40–1.21)	0.91 (0.47–1.79)
Socioeconomic Disconnection					
	Yes	8 (20.5)	31 (79.5)	1	1
	No	62 (17.9)	284 (82.1)	0.85 (0.37–1.93)	0.94 (0.35–2.52)
Monthly income (RMB)					
	≤3000	32 (21.9)	114 (78.1)	1	
	3001–5000	22 (16.3)	113 (83.7)	0.69 (0.38–1.27)	0.62 (0.30–1.28)
	>5000	16 (15.4)	88 (84.6)	0.65 (0.33–1.26)	0.61 (0.26–1.40)
Length of stay in Shenzhen (year)					
	>2	14 (10.8)	116 (89.2)	1	1
	1–2	11 (19.6)	45 (80.4)	2.03 (0.86–4.79)	2.34 (0.81–6.78)
	<1	45 (22.6)	154 (77.4)	2.42 (1.27–4.62)*	2.80 (1.30–6.05)*
Having vaginal sexual intercourse					
	Yes	48 (18.5)	212 (81.5)	1	1
	No	22 (17.6)	103 (82.4)	0.94 (0.54–1.65)	0.83 (0.41–1.68)
Age at first anal sexual intercourse (year)					
	≥18	50 (15.5)	272 (84.5)	1	1
	<18	20 (31.7)	43 (68.3)	2.53 (1.38–4.66)*	2.78 (1.29–5.89)*
Having a fixed homosexual partner					
	Yes	53 (19.5)	219 (80.5)	1	1
	No	17 (15.0)	96 (85.0)	0.73 (0.40–1.33)	0.74 (0.35–1.54)
Number of anal sexual partners in the past six months					
	≤2	22 (13.8)	137 (86.2)	1	1
	3–5	29 (26.6)	80 (73.4)	2.26 (1.21–4.19)*	2.54 (1.19–5.40)*
	>5	19 (16.2)	98 (83.8)	1.21 (0.62–2.35)	1.17 (0.52–2.61)
Roles in anal sexual intercourse					
	Insertive	13 (8.0)	149 (92.0)	1	1
	Receptive	29 (37.2)	49 (62.8)	6.78 (3.27–14.07)*	6.87 (3.02–15.61)*
	Both	26 (19.8)	105 (80.2)	2.84 (1.39–5.78)*	3.65 (1.64–8.09)*
Always using condom in anal sexual intercourse in the past six months					
	Yes	19 (11.4)	148 (88.6)	1	1
	No	48 (24.0)	152 (76.0)	2.46 (1.38–4.38)*	2.13 (1.08–4.19)*
HIV–related knowledge					
	<6	50 (18.5)	220 (81.5)	1	1
	≥6	20 (17.4)	95 (82.6)	0.92 (0.52–1.84)	0.91 (0.47–1.79)

*P < 0.05

### Factors associated with HIV and syphilis co-infection relative to mono-infection

Univariable logistic regression analyses indicated that having lived in Shenzhen for less than one year (OR = 2.18, 95% CI = 1.09–4.37) and playing exclusively receptive role in anal sexual intercourse (OR = 2.48, 95% CI = 1.15–5.34) was associated with HIV and syphilis co-infection, relative to the mono-infected individuals. Multivariable logistic regression analysis indicated that, compared with the mono-infected individuals, being unmarried (aOR = 2.47, 95% CI = 1.03–5.89) and playing exclusively receptive (aOR = 2.44, 95% CI = 1.04–5.73) role in anal sexual intercourse were associated risk factors for HIV and syphilis co-infection (Table 3).

**Table 3:** Logistic regression analyses of factors associated with HIV and syphilis co-infection (compared with the mono-infected individuals)

Variable	Category	HIV and syphilis infection (%)		Crude OR (95% CI)	Adjusted OR (95% CI)
		Co-infected group	Mono-infected group		
Age (year)					
	<25	9 (22.5)	31 (77.5)	1	1
	≥25	61 (34.3)	117 (65.7)	1.80 (0.80–4.01)	2.15 (0.85–5.44)
Ethnicity					
	Han	64 (31.5)	139 (68.5)	1	1
	Other minorities	6 (40.0)	9 (60.0)	1.45 (0.49–4.24)	1.29 (0.38–4.32)
Marital status					
	Married	13 (23.6)	42 (76.4)	1	1
	Unmarried	57 (35.0)	106 (65.0)	1.74 (0.86–3.50)	2.47 (1.03–5.89)*
Educational level					
	≤Junior middle school	24 (31.6)	52 (68.4)	1	1
	>Junior middle school	46 (32.4)	96 (67.6)	1.04 (0.57–1.89)	1.17 (0.59–2.35)
Socioeconomic Disconnection					
	Yes	8 (38.1)	13 (61.9)	1	1
	No	62 (31.5)	135 (68.5)	0.75 (0.29–1.89)	0.72 (0.25–2.09)
Monthly income (RMB)					
	≤3000	32 (33.0)	65 (67.0)	1	1
	3001–5000	22 (31.4)	48 (68.6)	0.93 (0.48–1.80)	1.00 (0.47–2.13)
	>5000	16 (31.4)	35 (68.6)	0.93 (0.45–1.92)	0.79 (0.33–1.93)
Length of stay in Shenzhen (year)					
	>2	14 (20.9)	53 (79.1)	1	1
	1–2	11 (39.3)	17 (60.7)	2.45 (0.94–6.40)	1.66 (0.56–4.97)
	<1	45 (36.6)	78 (63.4)	2.18 (1.09–4.37)*	1.34 (0.58–3.09)
Having vaginal sexual intercourse					
	Yes	48 (33.8)	94 (66.2)	1	1
	No	22 (28.9)	54 (71.1)	0.80 (0.44–1.46)	0.55 (0.26–1.19)
Age at first anal sexual intercourse (year)					
	≥18	50 (29.9)	117 (70.1)	1	1
	<18	20 (39.2)	31 (60.8)	1.51 (0.79–2.89)	1.50 (0.69–3.25)
Having a fixed homosexual partner					
	Yes	53 (35.6)	96 (64.4)	1	1
	No	17 (24.6)	52 (75.4)	0.59 (0.31–1.13)	0.63 (0.30–1.35)
Number of anal sexual partners in the past six months					
	≤2	22 (27.5)	58 (72.5)	1	1
	3–5	29 (41.4)	41 (58.6)	1.87 (0.94–3.69)	2.03 (0.86–4.77)
	>5	19 (27.9)	49 (72.1)	1.02 (0.50–2.10)	0.92 (0.37–2.28)
Roles in anal sexual intercourse					
	Insertive	13 (20.6)	50 (79.4)	1	1
	Receptive	29 (39.2)	45 (60.8)	2.48 (1.15–5.34)*	2.44 (1.04–5.73)*
	Both	26 (34.2)	50 (65.8)	2.00 (0.92–4.33)	2.40 (0.99 –5.79)
Always using condom in anal sexual intercourse in the past six months					
	Yes	19 (28.8)	47 (71.2)	1	1
	No	48 (35.0)	89 (65.0)	1.33 (0.71–2.53)	1.19 (0.55–2.57)
HIV–related knowledge					
	<6	50 (31.6)	108 (68.4)	1	1
	≥6	20 (33.3)	40 (66.7)	1.12 (0.63–1.81)	1.18 (0.59–2.35)

*P < 0.05

## Discussion

The findings of this cross-sectional study provide information about the prevalence of HIV, syphilis, and their co-infection, as well as factors associated with HIV and syphilis co-infection among ncMSM attending the STD clinic in Shenzhen, China.

The prevalence of HIV, syphilis and their co-infection among participants of this study was 24.2, 29.8 and 13.13%, respectively. The prevalence of HIV and syphilis found among ncMSM attending the STD clinic in this study were both higher than those found among the general MSM in the same area (3.3–5.3% for HIV and 10.5–14.3% for syphilis) [21, 41, 42]. In addition, the prevalence (13.13%) of HIV and syphilis co-infection found among ncMSM attending the STD clinic in this study was also higher than that (2.7%) reported in a meta-analysis among MSM in China [9], and also higher than that (1.5%) reported among MSM in 61 cities of China [43]. Different characteristics of study participants may influence the prevalence of these infections. For example, it is well-established that the prevalence of STDs among MSM attending the STD clinic was much higher than that observed among the general MSM [21]. Also influencing the prevalence of these infections among MSM could be the variations in population structures and economic development among cities from where studies select MSM samples [44]. As reported in previous studies, the prevalence of HIV and syphilis was higher in cities with larger floating populations and in more developed cities [44, 45]. Shenzhen is the first special economic zone in China with 87.0% of its total population being internal migrants [22]. Thus, the prevalence of HIV and syphilis co-infection in this city might be higher than that in cities with smaller floating populations or in less developed cities.

Among the socio-demographic variables of interest, this study found that marital status was independently associated with HIV and syphilis co-infection, when compared with HIV or syphilis mono-infection. Although a few studies have shown that marital status was related to either HIV or syphilis [46–48], none have reported a significant correlation between marital status and HIV and syphilis co-infection. Thus, the result found in this study that unmarried ncMSM were more likely to be co-infected with HIV and syphilis, adds to the existing knowledge of HIV and syphilis co-infection among MSM. It could be deduced that the different sexual behaviors found between married and unmarried MSM [49], could explain the significant difference in the likelihood of HIV and syphilis co-infection among the married and unmarried ncMSM in this study.

While this study found no significant association between age and HIV and syphilis co-infection among ncMSM, many previous studies indicated that age significantly played an important role in HIV and syphilis infections, as well as in the HIV and syphilis co-infection [16, 23, 50]. A possible reason for the insignificant relationship between age and HIV and syphilis co-infection in this study could be that the proportion of participants aged less than 25 was relatively low, accounting for only 16.1% in co-infected and non-infected group, and only 18.3% in co-infected and mono-infected group. Therefore, the discrimination of age between co-infected and non-infected ncMSM, and between co-infected and mono-infected ncMSM was probably too small to be detected.

Furthermore, the results of this study indicated that, when compared with non-infected ncMSM, ncMSM having lived in Shenzhen for less than one year were more likely to be co-infected with HIV and syphilis. This result and the fact that migration has been shown to be an essential risk factor for HIV in many populations around the world [45, 51], are evidence to suspect that migrant ncMSM in Shenzhen are at higher risk for HIV and syphilis co-infection and, hence, need special attention in HIV and syphilis prevention.

Although some studies observed that HIV-related knowledge significantly correlated with HIV and syphilis infections [40, 52], this study, however, found no association between them. This may be due to an even promotion of HIV-related knowledge by the local government or communities. Therefore, the participants were probably given the same opportunity to be almost equally knowledgeable of HIV transmission. It is necessary to declare here that promoting HIV-related knowledge could significantly reduce the prevalence of HIV-related infections [25].

Moreover, as in previous studies [53, 54], some risky sexual behaviors were found to be associated with HIV and syphilis co-infection in this study. In particular, when compared with non-infected ncMSM, those having first anal sexual intercourse before the age of 18, having 3 to 5 anal sexual partners in the past six months, playing exclusively receptive or both insertive and receptive roles in anal sexual intercourse, or not always use condom in anal sexual intercourse were more likely to be co-infected with HIV and syphilis. However, when compared with mono-infected ncMSM, only those playing exclusively receptive role in anal sexual intercourse were at higher risk for being co-infected with HIV and syphilis. Future studies with large sample sizes are needed to clarify the mechanism of HIV and syphilis co-infection and further elucidate why associated factors for HIV and syphilis co-infection in ncMSM were quite different when compared with the non-infected group and mono-infected group.

Certain limitations should be acknowledged. Firstly, the design of this study is cross-sectional, so causal inferences between associated factors and HIV and syphilis co-infection cannot be concluded. Future longitudinal studies with large sample sizes are warranted to clarify the casual inferences between them. Secondly, the convenience sampling method used for selecting participants and the relatively small sample size may reduce the generalizability of our findings to ncMSM attending the STD clinic in other cities. Thirdly, most of the study participants (≥80%) were aged at least 25, Han ethnicity, socio-economically connected and had experienced anal sexual debut aged at least 18, as a result of which, the statistical power to explore the association between each of these characteristics and HIV and syphilis co-infection among ncMSM might be lowered. Fourthly, though managed by well-trained investigators, this survey was partly retrospective. Recall bias might exist when the respondents answered some questions, such as the age at first anal sexual intercourse and the number of anal sexual partners in the past six months. Finally, by including only participants aged at least 18, the results of this study might not be applicable to ncMSM aged less than 18. Considering these limitations, caution must be exercised when extrapolating the findings of this study.

Despite the preceding limitations, this study has quite a few strengths, as well as implications for service providers. Firstly, to the best of our knowledge, this is the first study to explore the prevalence of HIV and syphilis co-infection and associated factors among ncMSM attending the STD clinic. Therefore, its findings are fundamental in the prevention of HIV and syphilis co-infection among ncMSM. Secondly, the high prevalence of HIV and syphilis co-infection found in this study suggests that the service providers need to intensify efforts to prevent the spread of HIV and syphilis among ncMSM attending the STD clinic. Thirdly, a wide array of exploratory factors was considered in this study, which not only included socio-demographic characteristics and risky sexual behaviors, but also HIV-related knowledge. Therefore, the effects brought by potential confounding when analyzing the data and interpreting the results were substantially reduced. Finally, in this study, the finding that associated factors for HIV and syphilis co-infection in ncMSM were quite different when compared with the non-infected group and mono-infected group strongly urges the service providers to accordingly adjust surveillance methods for HIV and syphilis co-infection with respect to the intensified intervention strategies targeting at the non-infected and mono-infected ncMSM, in order to reduce HIV and syphilis co-infection among MSM.

## Conclusions

In summary, HIV and syphilis co-infection is quite prevalent among the study participants in Shenzhen. Given the different associated factors found in this study for HIV and syphilis co-infection among ncMSM relative to non-infected and mono-infected ncMSM, integrated HIV and syphilis intervention strategies, specifically targeting at the non-infected and mono-infected ncMSM, are needed to reduce HIV and syphilis co-infection among MSM. Future studies with large sample sizes are needed to clarify the mechanism of HIV and syphilis co-infection in MSM.

## Abbreviations

95% CI: 95% confidence interval
aOR: Adjusted odds ratio
CCDC: Center for chronic disease control
CDC: Center for disease control and prevention
ELISA: Enzyme-linked immunosorbent assay
HIV: Human immunodeficiency virus
MBs: Money boys
ncMSM: Non-commercial men who have sex with men
OR: Odds ratio
SD: Standard deviation
STD: Sexually transmitted disease
TRUST: Tolulized red unheated serum test
